# Investigation of the correlation between cardiac parameters and aortic diameter in patients with ascending aortic aneurysm

**DOI:** 10.1186/s43044-022-00238-0

**Published:** 2022-01-07

**Authors:** Mustafa Etli, Seda Avnioglu, Halil Yilmaz, Oguz Karahan

**Affiliations:** 1 Department of Cardiovascular Surgery, Medical School of Aladdin Keykubat University, Alanya-Antalya, Turkey; 2 Department of Physiotherapy and Rehabilitation, Kozaklı Vocational School of Hacı Bektas Veli University, Nevsehir, Turkey

**Keywords:** Ascending aortic aneurysm, Diameter, Cardiac volume, Echocardiography, Computed tomography

## Abstract

**Background:**

Aortic aneurysms (AA) are enlargement of the aorta silently until diagnosing, not detectable on physical examination, and usually incidentally discovered during radiologic scanning for other reasons. It can get bigger sizes and can result in life-threatening outcomes if not detected early on. In this study, we aimed to determine the relationship between ascending aortic diameter and cardiac parameters that can be detected with tomography or/and echocardiography. Newly diagnosed (*n*: 85) ascending AA patients and healthy individuals (*n*: 86) who have not any thoracic pathology in computed tomography (CT) scans included to the study. Echocardiographically determined left atrial dimension (LAD), left ventricular end-diastolic diameter (LVDd), left ventricular end-systolic diameter (LVDs), left ventricular ejection fraction (LVEF) and the left ventricular posterior wall thickness (LVPWd) values of each patient were recorded. The thorax diameters, ascending aorta diameters and cardiac volume values recorded from CT scans. The obtained findings were statistically compared.

**Results:**

Positive correlation was found between aortic diameter and aging (*p*: 0.000). Increased thorax diameter and cardiac volume values were detected in ascending AA cases (*p* < 0.05). It was found to be ascending aortic diameter was positively correlated with thorax diameter and cardiac volume (0.50 < *r* ≤ 0.70) values and higher aortic diameter, cardiac volume, thorax diameter values were detected in male individuals when compared with the female gender. There was no significant correlation between LVEF, LVDd, and LVDs values and aortic diameter.

**Conclusions:**

Cardiac volume and thorax diameter were found as strongly correlated with the diameter of the ascending aorta. The clarifying of these parameters with larger cohorts might be beneficial for the estimation of the progression of ascending AA.

## Background

Aortic aneurysm (AA) is a multifactorial disease that is correlated with progressive weakening of the aortic wall [1]. Atherosclerosis, high blood pressure, genetic and congenital (bicuspid aortic valve) factors have been reported as associated risk factors for disease progression [2]. In particular, ascending AA is a highly mortal and morbid pathology by aging, though relatively few reports have included it, in comparison to abdominal AA [2, 3]. Ascending AA is usually diagnosed incidentally in echocardiography examinations without aortic complaints. Thus, despite well-known high mortal risks of aortic rupture and dissection in ascending AA patients, clinicians have not been sufficiently informed about optimal management of patients with a dilated ascending aorta [3]. Abdel Razek et al. presented a comprehensive review of computed tomography (CT) angiography and magnetic resonance (MR) in the evaluation of the main cardiac and vascular structures. They declared that CT angiography provides good anatomic visualization for vascular beds and surrounding tissue jointly. Therefore, CT and MR angiography can be suggested as beneficial diagnostic imaging methods for these structures [4].

The literature includes a number of reports regarding aortic diameters and mortality risks. The management of ascending AA is also related with the diameter of the aorta and the decision to opt for surgery is dependent on this diameter [3, 5]. Although there are some studies reporting on the enlargement of aorta or presenting of shear stress on aortic wall, related cardiac findings have not been sufficiently clarified [1]. Some cardiac factors have been reported as significant risk factors of ascending AA, such as coronary heart disease and atrial fibrillation [5]. However, the thoracic or cardiac diameters in ascending AA have not been investigated in previous studies and so in the present study, we aimed to investigate these, the changes of cardiac volume in patients with ascending AA, as well as the possible correlation of cardiac volume and aortic diameter.

## Methods

Ethical approval was received from the local ethical committee of the University. All steps of study were conducted according to the principles of the Helsinki Declaration and in adherence to the local guidelines for good clinical practice. The patients, who were newly diagnosed with isolated ascending aorta dilatation without any complications, were included to the study. Exclusion criteria were determined as follows: accompanying cardiac disease, valve disease in echocardiogram, bicuspid aorta, vascular operation history, peripheral vascular disease, pulmonary embolism, pulmonary infection, chronic obstructive lung disease and already known hereditary or immune disease and drug usage history resulting from any chronic disease. Individuals were selected from recorded computed tomography scans used to diagnose AA incidentally.

### Patient selection and group creation

This study is a non-randomized controlled trial. The population of this study consists of patients incidentally diagnosed ascending AA patients without any complaints in routine control. To estimate sample collection with Gpower 3.1.9.4 program was used. It was done by power analysis using software. The power value detection of left ventricular ejection fraction (LVEF) was made by depending on the reference article [6].

Echocardiographic findings and Computed tomography (CT) angiography records were scanned retrospectively. After preparing a participant pool from the tomography records, initially unsuitable individuals were excluded to the study, in accordance with the exclusion criteria. The patients with isolated ascending aorta dilatation were designated as the study group (n: 85) and individuals who had normal aorta diameters were designated as the control group (n: 85). Ascending aorta diameters, thorax diameters, left and right lung volumes and cardiac volumes were measured in CT angiograms from all participants.

### Computed tomography measurements

**Fig. 1.**
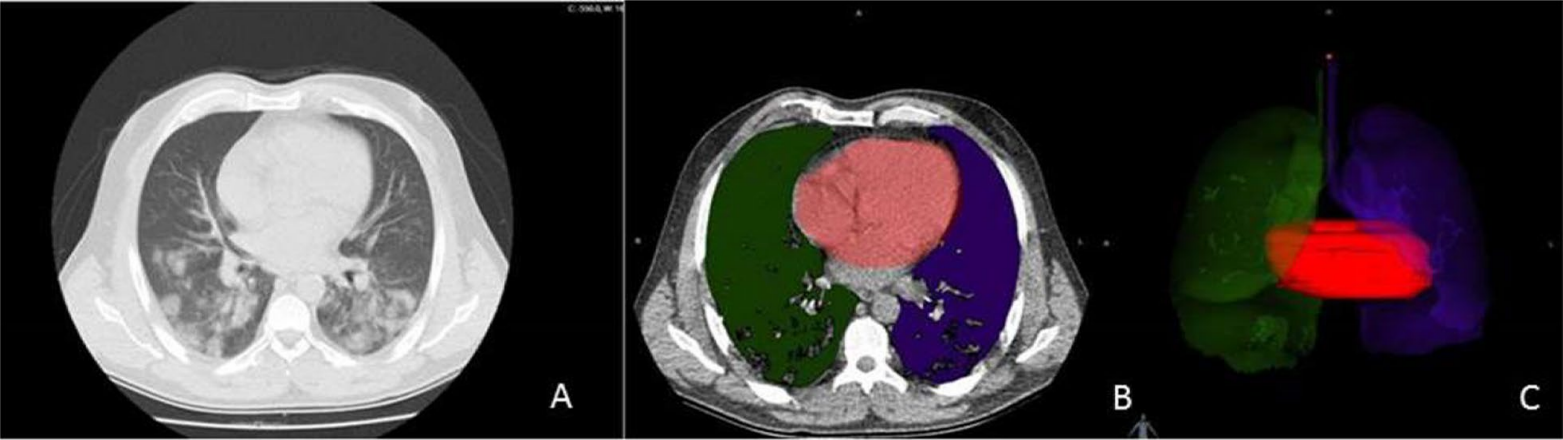
3D planning of thoracic organs by Myrian® software: **A** The transverse plane of thorax tomography, **B** the color scale of left (blue) and right (green) lung, and heart (pink), **C** 3-D plan of thorax tomography by Myrian® software

CT angiography scans were done by 16-detector CT device (Toshiba Alexion™/Advance, Toshiba Medical Systems Corporation Nashu, Japan). The thoracic structures were evaluated at 1 mm thickness, 120 kVp, 50–65 mAs, 0.938 pitch, 0.75 s rotation time, 16 × 1 collimation, matrix 512 × 512 and 250 × 300 mm FOV. Three dimensional (3D) multiplanar image reformation and maximum density projections were performed by on a radiology workstation (Sectra Workstation IDS 7, Linköping, Sweden). The morphological parameters such as the diameter of the ascending aorta, cardiac volume and thorax diameter were calculated. These calculations were performed on 3D plans by using available software program Intrasense Myrian® (Myrian; Intrasense, Montpellier, France). This application is the process of defining the anatomical structures of the patient, coloring the area of interest and lifting them into three dimensions. In our study, we firstly defined the lung and heart regions on the CT images that we transferred to the Myrian® software, and then we measured the thorax diameter together with the cardiac volume measurement from the program, by making the appropriate coloring (Fig. 1).

### Echocardiographic evaluation

Routine echocardiographic evaluations were conducted by an experienced echocardiographer, based on the recommendations of the American Society of Echocardiography and the European Association of Cardiovascular Imaging [7]. All examinations were made by left parasternal approach using a Philips iE33 (S5-1 probe, iE33, Philips Medical Systems, Andover, MA) ultrasound device, with a 3.5-MHz ultrasound probe. The determined left atrial dimension (LAD), left ventricular end-diastolic diameter (LVDd), left ventricular end-systolic diameter (LVDs), left ventricular ejection fraction (LVEF) and the left ventricular posterior wall thickness (LVPWd) values of each patient were recorded.

### Statistical analysis

Data were then evaluated and expressed in terms of inter-observer agreement in pairs. The statistical analyses were made with by using a software program (IBM SPSS 22.0). Normal distribution weight analyses were made by evaluating five parameters (kurtosis-skewness, Histogram, Q-Q plots, Std/Mean, Shapiro Wilk Test) from the data obtained. The data that took three points from these five parameters were considered as normally distributed. The Independent Samples T Test was used for binary group analysis as parametric assessment test, the Pearson Correlation test was used for correlation analysis, and the Pearson Chi-Square Test was used for frequency analysis: *p* < 0.05 was considered as statistically significant.  

## Results

In our study, it was determined that Chi-square analysis, men (35.8%) had more aneurysms than women (14.0%) (*p* < 0.01) (Table 1). The morphological findings and ages of groups obtained are summarized in Table 2. The Independent Samples T Test revealed that the ascending AA group had higher age ranges (64.70 ± 12.87 years) when compared with the control group (49.95 ± 16.37 years) (*p* < 0.001). Additionally, the diameter of the ascending aorta, the diameter of the thorax and the cardiac volume was found to be markedly higher in the ascending AA group (*p* < 0.05). These values were analyzed in regards to gender (Table 3) and there were no differences between male and female gender in regards to age distribution (p: 0.165). On the contrary, the diameter of the ascending aorta and of the thorax, as well as cardiac volume values were found to be significantly higher (Table 2) in the male gender (*p* < 0.05).

**Table 1:** Relation between gender and aneurysm (Pearson Chi-square)

Aneurysm	Gender			Pearson Chi-Square Test
	Female	Male	Total	**Value = 17.394^a^ *p* = 0.000
Aneurysm	24 (14.0%)	61 (35.7%)	85 (49.7%)	
Control	51 (29.9%)	35 (20.4%)	86 (50.3%)	
Total	75 (43.9%)	96 (56.1%)	171 (100%)	

**Difference is significant at the 0.01 level (2-tailed)

^a^Positive relation between Man gender and Aneurysm (*p* < 0.01)

**Table 2:** Independent samples test morphologic and demographic variables by aneurysm

	*N*	Mean	Sig. (*p*)
Age			
Patient		*64.70 ± 12.87	0.000
Control		49.95 ± 16.37	
Ascending aorta diameter			
Patient		*45.37 ± 4.22	0.000
Control		20.74 ± 2.15	
Cardiac volume			
Patient		*520.61 ± 114.87	0.000
Control		358.84 ± 74.42	
Thorax diameter			
Patient		*417.58 ± 72.58	0.000
Control		347.44 ± 65.00	

Parametric data’s were shown as Mean ± Standard deviation (Std) t test was used for comparisons of two independent groups

*The difference between groups is statistically significant at 99.9% confidence (*p* < 0.001)

**Table 3:** Independent samples test morphologic and demographic variables by gender

	*N*	Mean	Sig. (*p*)
Age			
Female	75	55.36 ± 16.21	0.165
Male	95	58.88 ± 16.54	
Ascending aorta diameter			
Female	75	28.74 ± 11.89	0.000
Male	95	*36.47 ± 12.49	
Cardiac volume			
Female	75	380.18 ± 111.25	0.000
Male	95	*485.65 ± 118.28	
Thorax diameter			
Female	75	325.93 ± 50.11	0.000
Male	95	*426.02 ± 65.29	

Parametric data’s were shown as Mean ± Standard deviation (Std) t test was used for comparisons of two independent groups

*The difference between groups is statistically significant at 99.9% confidence (*p* < 0.001)

**Fig. 2:**
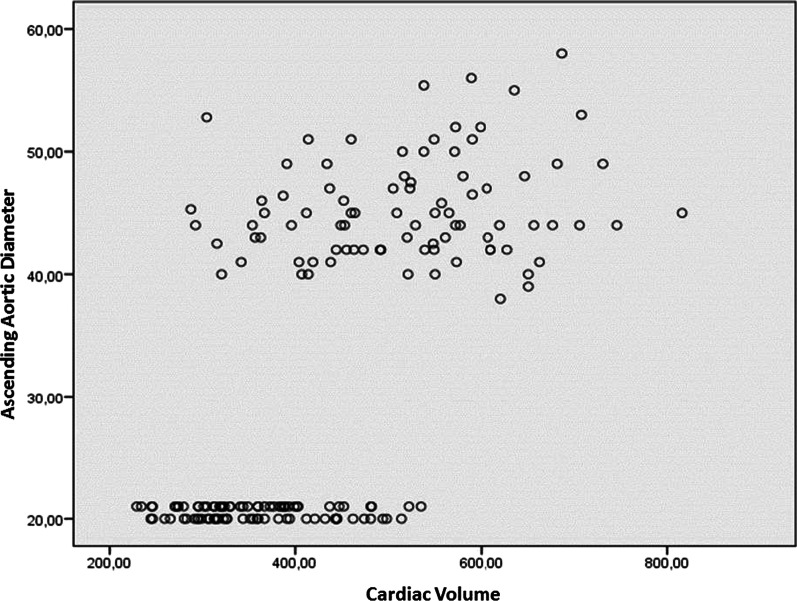
The correlation between Aortic diameter and Cardiac volume

The Pearson Correlation Value (r) evaluation for correlation analyze was made according to previous literature [8]. Considering the correlation between the morphological analysis performed, heart volume (Fig. 2) was found to be moderately positive correlated with both aortic diameter (0.50 < *r* ≤ 0.70, *p* < 0.001) and thorax diameter (0.50 < *r* ≤ 0.70, *p* < 0.01) (Table 4).

**Table 4:** Pearson correlations morphologic and demographic variables

	Age	Ascending aorta diameter	Cardiac volume	Thorax diameter	A. Aorta
Age					
*r*	1	0.445^B^	0.0360^B^	0.325^B^	0.066^A^
Sig. (*p*)*		0.000	0.000	0.000	0.542
Ascending aorta diameter					
*r*	0.445^B^	1	0.648^C^	0.463^B^	0.488^B^
Sig. (*p*)*	0.000		0.000	0.000	0.000
Cardiac volume					
*r*	0.360^B^	0.648^C^	1	0.589^C^	0.163^A^
Sig. (*p*)*	0.000	0.000		0.000	0.132
Thorax diameter					
*r*	0.325^B^	0.463^B^	0.589^C^	1	− 0.021^A^
Sig. (*p*)*	0.000	0.000	0.000		0.849
A. Aorta					
*r*	0.066^A^	0.488^B^	0.163^A^	− 0.021^A^	1
Sig. (*p*)*	0.542	0.000	0.132	0.849	

*Correlation is significant at the 0.001 level (2-tailed)

^A^No correlation, ^B^Low positive correlation, ^C^Moderate positive correlation

The correlations analyze revealed high positive correlation between LVDd and LVDs (0.70 < *r* ≤ 0.90, *p* < 0.001) (Table 5). A weak negative correlation was found between heart volume and LVEF value (-0.50 < *r* ≤ -0.30, *p* < 0.01), on the other hand it was a low positive correlation between heart volume-LVDs (0.50 < *r* ≤ 0.30, *p* < 0.01) and heart volume-LVDd values (0.50 < *r* ≤ 0.30, *p* < 0.01) (Table 6).

**Table 5:** Pearson correlations eco variables

	IVSd	LVDd	LVPWd	LVDs	LVEF	LAD
IVSd						
*r*	1	*0.420^B^	− 0.073^A^	*0.341^B^	*− 0.342^B^	0.128^A^
Sig. (*p*)		0.001	0.566	0.006	0.006	0.346
LVDd						
*r*	*0.420^B^	1	0.003^A^	**0.821^C^	*− 0.334^B−^	0.054^A^
Sig. (*p*)	0.001		0.980	0.000	0.007	0.691
LVPWd						
*r*	− 0.073^A^	0.003^A^	1	− 0.030^A^	0.031^A^	− 0.077^A^
Sig. (*p*)	0.566	0.980		0.813	0.806	0.574
LVDs						
*r*	*0.341^B^	**0.821^C^	− 0.030^A^	1	**− 0.440^B^	− 0.052^A^
Sig. (*p*)	0.006	0.000	0.813		0.000	0.706
LVEF						
*r*	*− 0.342^B−^	*− 0.334^B−^	0.031^A^	**− 0.440^B−^	1	0.108^A^
Sig. (*p*)	0.006	0.007	0.806	0.000		0.424
LAD						
*r*	0.128^A^	0.054^A^	− 0.077^A^	− 0.052^A^	0.108^A^	1
Sig. (*p*)	0.346	0.691	0.574	0.706	0.424	

LAD: Left atrial dimension, LVDd: Left ventricular end-diastolic diameter, LVDs: Left ventricular end-systolic diameter,

LVEF: Left ventricular ejection fraction, LVPWd: Left ventricular posterior wall thickness, IVSd: Interventricular septum at end diastole

*Correlation is significant at the 0.01 level (2-tailed)

**Correlation is significant at the 0.001 level (2-tailed)

^A^Very week or Negligible correlation, ^B^Low positive correlation, ^B−^Low negative correlation, ^C^High positive correlation

**Table 6:** Pearson correlations between morphologic and echocardiography variables

	Age	Ascending aorta diameter	Cardiac volume	Thorax diameter
IVSd				
*r*	**0.333^B^	0.036^A^	*0.265^A^	0.137^A^
Sig. (*p*)	0.007	0.780	0.034	0.281
LVDd				
*r*	0.146^A^	0.225^A^	**0.332^B^	*0.299^A^
Sig. (*p*)	0.253	0.077	0.008	0.017
LVPWd				
*r*	− 0.72^A^	0.113^A^	0.101^A^	0.157^A^
Sig. (*p*)	0.574	0.372	0.427	0.216
LVDs				
*r*	0.074^A^	0.220^A^	**− 0.383^B−^	− 0.186^A^
Sig. (p)	0.019	0.341	0.004	0.108
LVEF				
*r*	− 0.269^A^	− 0.111^A^	**− 0.324^B−^	− 0.186^A^
Sig. (*p*)	0.019	0.341	0.004	0.108
LAD				
*r*	− 0.188^A^	− 0.016^A^	0.016^A^	− 0.149^A^
Sig. (*p*)	0.162	0.904	0.907	0.269

LAD: left atrial dimension, LVDd: left ventricular end-diastolic dimension, LVDs: left ventricular end-systolic dimension, LVEF: left ventricular ejection fraction, LVPWd: left ventricular posterior wall thickness,, IVSd: Interventricular septum at end diastole

*Correlation is significant at the 0.05 level (2-tailed)

**Correlation is significant at the 0.01 level (2-tailed)

^A^Very week or Negligible correlation, ^B^ Low positive correlation, ^B−^Low negative correlation

## Discussion

To the best of our knowledge, this is the first comprehensive study that compares ascending aorta diameter and thoracic diameters and volumes in normal and asymptomatic ascending AA cases. The main findings of our study can be briefly summarized as follows: ascending AA is related to aging, increased thorax diameter and cardiac volume values. Ascending aortic diameter was positively correlated with thorax diameter and cardiac volume values, and higher aortic diameter, cardiac volume, thorax diameter values were detected in male individuals, when compare with female gender. Despite statistically insignificant, higher LVDd and LVDs values were detected in ascending AA cases. However, lower correlation was found between cardiac volume and these echocardiographic parameters.

Ascending AA are highly mortal pathologies if they are not diagnosed and treated in a timely manner. Because of good clinical outcomes after surgery of ascending AA, the management of the disease and understanding the predictive indicators are critical [9]. The management of the disease mainly depends on aortic dimensions and the enlargement of the aorta should be followed up clinically. However, the enlargement at any time or the rupture risk for the borderline disease cannot be determined. [1, 5]. Wolak et al. suggested that body surface area (BSA), gender and age are important predictors for aortic dimensions [9]. They found as there is a relation between aging and aortic diameter and they determined that gender is a significant predictor only when interacting with age. BSA was found to be a stronger determinant factor for aortic dimensions when compared with body mass index (BMI) in their study [9]. It was reported that the diameter of the aorta increases with age. In younger ages, the diameters remains fixed, but with age increase, the diameters of the aorta appears to be significantly affected by age and gender [10, 11]. Rylski et al. reported that the female gender is associated with smaller aortic dimensions at a young age, which may help protect against aortic dissection. They also report that the aortic enlargement and the risk of aneurysm is lower in the female gender at a younger age, and though the female aorta is smaller than the male aorta, aortic growth dynamics throughout life are greater in women than in men [12]. According to our results, ascending AA has a higher incidence in men when compare with women, and men have higher aortic diameters. Additionally, we found that ascending AA patients have a higher age when compared with individuals who had normal aortic diameters. In a morphological study, Mao et al. found that men have higher thorax diameters as well as aortic diameter, when compare with female gender [11]. In a cross-sectional study by Ray et al., it was reported that thorax and heart diameters as well as aortic diameters, are found to be higher in the male gender [13]. Similarly, our results revealed higher thorax diameters and cardiac volume values in males.

There are very few publications on the size of the aorta and its correlation with heart size. Another result from a Ray et al. study found a positive correlation between aortic diameter, heart diameter and transverse thoracic diameter [13]. Moreover, strong positive correlation of aortic arch diameter with chest and heart diameters was shown in other previous reports [14]. We found that ascending aortic diameter was positively correlated with thorax diameter and cardiac volume values. In aortic dissection patients, it was previously found that aortic diameter is related with cardiac hypertrophy, and in the same study, marked correlation was found between aortic diameter and left ventricle mass index (LVMI) [15]. Similarly, Erdogan et al. found a statistical relationship between LVMI and ascending aorta size, whereas on the other hand, they found significant difference between patients with dilated ascending aorta and normal populations, in regards to LVDd, LVPWd, LVEF, and LAD [16]. Similarly, a significant relationship was reported by Iarussi et al. between aortic diameter and cardiac parameters, such as LVDd, LVDs and LVMI index, in an analysis of aortic dissection patients [17]. Although statically insignificant, we found higher LVDd and LVDs values in ascending AA patients.

## Conclusions

In conclusion, according to our results, thorax diameter and cardiac volume were positively correlated with ascending aorta diameter. Furthermore, male gender and age are both factors affecting the diameter of the ascending aorta. It appears that cardiac volume is the main determinant for the ascending aorta diameter. We believe that the mechanism of action and progression of the disease can be better understood by investigating and clarifying the differences in cardiac parameters, between normal and AA cases.

### Limitations of study

This study has several limitations. First, this was a single-center study and the normal population that was used as a control population for ascending AA consisted in a younger age group. Secondly, we extracted our results from a relatively small sample size. Finally, the obtained results were produced from retrospective data. It would be beneficial to have the results of our study confirmed in larger prospective series.

## Data Availability

The data used to support the findings of this study are included within the article.

## Abbreviations

AA: Aortic aneurysm
CT: Computed tomography
LAD: Left atrial dimension
LVDd: Left ventricular end-diastolic diameter
LVDs: Left ventricular end-systolic diameter
LVEF: Left ventricular ejection fraction
LVPWd: Left ventricular posterior wall thickness
IVSd: Interventricular septum at end diastole
